# Aquila_stLFR: diploid genome assembly based structural variant calling package for stLFR linked-reads

**DOI:** 10.1093/bioadv/vbab007

**Published:** 2021-06-16

**Authors:** Yichen Henry Liu, Griffin L Grubbs, Lu Zhang, Xiaodong Fang, David L Dill, Arend Sidow, Xin Zhou

**Affiliations:** 1 Department of Computer Science, Vanderbilt University, Nashville, TN 37235, USA; 2 Department of Biomedical Engineering, Vanderbilt University, Nashville, TN 37235, USA; 3 Department of Computer Science, Hong Kong Baptist University, Kowloon Tong, Hong Kong; 4 BGI Tech, BGI Genomics, Shenzhen 518083, China; 5 Department of Computer Science, Stanford University, Stanford, CA 94305, USA; 6 Department of Pathology, Stanford University, Stanford, CA 94305, USA

## Abstract

**Motivation:**

Identifying structural variants (SVs) is critical in health and disease, however, detecting them remains a challenge. Several linked-read sequencing technologies, including 10X Genomics, TELL-Seq and single tube long fragment read (stLFR), have been recently developed as cost-effective approaches to reconstruct multi-megabase haplotypes (phase blocks) from sequence data of a single sample. These technologies provide an optimal sequencing platform to characterize SVs, though few computational algorithms can utilize them. Thus, we developed Aquila_stLFR, an approach that resolves SVs through haplotype-based assembly of stLFR linked-reads.

**Results:**

Aquila_stLFR first partitions long fragment reads into two haplotype-specific blocks with the assistance of the high-quality reference genome, by taking advantage of the potential phasing ability of the linked-read itself. Each haplotype is then assembled independently, to achieve a complete diploid assembly to finally reconstruct the genome-wide SVs. We benchmarked Aquila_stLFR on a well-studied sample, NA24385, and showed Aquila_stLFR can detect medium to large size deletions (50 bp–10 kb) with high sensitivity and medium-size insertions (50 bp–1 kb) with high specificity.

**Availability and implementation:**

Source code and documentation are available on https://github.com/maiziex/Aquila_stLFR.

**Supplementary information:**

Supplementary data are available at *Bioinformatics Advances* online.

## 1 Introduction

Short-read sequencing has had a major influence on human genetic studies; however, identifying structural variants (SVs ≥ 50 bp) has been a limitation. Recently developed sequencing technologies, including 10X Genomics, TELL-Seq and single tube long fragment reads (stLFR) offer promise for large-scale ‘perfect genome’ assembly (McElwain *et al.*, 2017; Zheng *et al.*, 2016) as they combine low sequencing error and long-range contiguity. The 10X Genomics Chromium system is based on Microfluidic technology, where DNA long fragments are allocated to millions of compartments and attached with particular barcodes. The reactions of stLFR happen in a single tube and then unique barcodes are attached on the surface of microbeads. stLFR enables co-barcoding of over 8 million 20–300 kb genomic DNA fragments, and these long-range fragments enable efficient phasing, resulting in long phase block N50 (Wang *et al.*, 2019). The long-range information of 10X linked-reads allows easier detection of SVs, *de novo* mutations, and haplotype phasing (Zheng *et al.*, 2016; Zhou *et al.*, 2018). Taking advantage of these linked-reads data to generate a diploid assembly and detect SVs requires the development of new algorithms. Aquila, developed recently for 10X linked-reads, achieved diploid assembly and reconstruction of genome-wide SVs with high sensitivity and accuracy (Zhou *et al.*, 2021). This reference-assisted, diploid assembly based approach incorporates the high information content of the reference to help partition linked-reads into parental haplotypes for local assembly, which enables comprehensive detection of variants spanning the target loci from diploid sequences (contigs). The recent patent dispute regarding 10X linked-reads necessitates taking advantage of other linked-read technologies. Thus, we develop Aquila_stLFR, which extends Aquila to the key characteristics of stLFR to identify SVs from diploid assembly with a higher accuracy compared to other methods such as GROC-SVs (Spies *et al.*, 2017) and NAIBR (Elyanow *et al.*, 2018). We also introduce a hybrid assembly mode, ‘Aquila_hybrid’, to enable combining both stLFR and 10X linked-reads data.

## 2 Methods

Aquila_stLFR is a reference-assisted, local *de novo* assembly pipeline (Supplementary Figure 1). The reference is used to allocate long fragment reads (LFRs) into haplotype-specific blocks, and local assembly is then performed within small chunks independently for both haplotype-specific blocks. The input files for Aquila_stLFR are a FASTQ file with paired short reads, a BAM file and a VCF file. To generate the BAM file, each sequence identifier line in the FASTQ file needs to contain the barcode identifier, starting with ‘BX:Z:’ (for instance, ‘BX:Z:540_839_548’ where 540_839_548 is the barcode identifier, Supplementary Figure 2). The BAM file generated by BWA-MEM with the ‘-C’ flag will then also include the extra field ‘BX:Z:’ for Aquila_stLFR to reconstruct LFRs (Supplementary Figure 3). Ideally, each individual LFR is attached to the same unique barcode and sequenced by short-read sequencing, so short reads with the same barcode could be linked together to form the original LFR (linked-read). Aquila_stLFR reconstructs all LFRs relying on this concept. However, it is still necessary to tackle the barcode deconvolution problem: each group of reads that share the same barcode is drawn from an unobserved set of fragments, since the ideal design of one unique barcode per LFR will not be achieved for all LFRs during the preparation of real sequencing libraries. stLFR technology labels all reads that originate from a small number of DNA molecules (20–300 kb in length) with the same molecular barcode. Aquila_stLFR applies an empirical boundary threshold (50 kb by default) to differentiate between two LFRs with the same barcode. For instance, if the distance between two successive reads with the same barcode is larger than this threshold, they will be assumed to be drawn from two different LFRs. In pilot studies, we tested 20, 40, 50, 60 and 80 kb for this boundary threshold parameter for both stLFR and 10X linked-reads. The choice of threshold had no significant impact on assembly and SV detection results.

After reconstructing all LFRs, Aquila_stLFR utilizes single nucleotide polymorphisms (SNPs) from the input VCF file to annotate each LFR with heterozygous SNPs and then relies on all pairs of heterozygous SNPs supported by different clusters of LFRs to apply a recursive clustering algorithm to finally partition LFRs into haplotype-specific blocks (Zhou *et al.*, 2021). Aquila_stLFR further cuts large haplotype-specific blocks into small chunks (100 kb by default). Finally, short reads that are drawn from LFRs are identified and reassembled locally by SPAdes (Bankevich *et al.*, 2012) within each small chunk. Because of the higher percentage of multi-mapped, 100 bp-length short reads in stLFR compared to the 150 bp-length short reads from 10X Genomics, it was necessary for stLFR sequencing to involve all short reads based on the barcode for local assembly instead of filtering out some reads like Aquila. Aquila_stLFR was thus designed to define and extract reads of each LFR from both the BAM and the original FASTQ instead of only the BAM file used in Aquila for 10X linked-reads. To achieve large contiguity, Aquila_stLFR iteratively concatenates assembled mini-contigs from small chunks into full-length contigs. To detect SVs from the diploid assembly, Aquila_stLFR finally performs a pairwise comparison between haploid contigs and reference by integrating Minimap2 (Li, 2018) and paftools (https://github.com/lh3/minimap2/tree/master/misc). Additionally, the ‘Aquila_hybrid’ mode applies an analogous concept to reconstruct long DNA fragments and generate the same data structure for long DNA fragments of both 10X and stLFR data (Supplementary Figure 1). Aquila_hybrid can combine both linked-reads data for diploid assembly. We have used both stLFR and 10X linked-reads for hybrid assembly and SV detection and present the results in the Supplementary file.

## 3 Results and discussion

In this study, we used the stLFR sequencing library for NA24385 from the Genome in A Bottle (GiAB) website (ftp://ftp-trace.ncbi.nlm.nih.gov/giab/ftp/data/AshkenazimTrio/HG002_NA24385_son/stLFR/), since we can evaluate SV (≥50 bp) detection by comparing Aquila_stLFR’s calls against the GiaB NA24385 SV benchmark callsets. This stLFR library has approximately 48X Illumina sequencing coverage, and the average inferred (reconstructed) DNA fragment (LFR) length is around 30 kb. To demonstrate the performance of SV calling for Aquila_stLFR, we have compared it against two other linked-reads SV callers: GROC-SVs and NAIBR (Table 1). After generating SV calls for NA24385 from all three methods, we used Truvari (https://github.com/spiralgenetics/truvari, parameters -p 0.1, -P 0.1, -r 200, –passonly) to compare SV calls with the benchmark callset from three different SV size range thresholds. In Table 1, we showed that Aquila_stLFR achieved sensitivity of 80.5% for deletions in the range of 50 bp–1 kb) and 63.5% for deletions in the range of 1 kb–10 kb, which were higher than the other two methods. NAIBR achieved a higher sensitivity of 82.8% for deletions larger than 10 kb. Since few insertions were found by GROC-SVs and NAIBR, we only performed insertion evaluation for Aquila_stLFR. Aquila_stLFR achieved a high specificity of 83.8% with a sensitivity of 23.4% for insertions in the range of 50 bp–1 kb. In conclusion, Aquila_stLFR is the first approach that effectively leverages the strengths of linked-read sequencing to enable medium to large SV discovery.

**Table 1.** Genome-wide deletion evaluation against GIAB NA24385 benchmark

		Aquila_stLFR	GROC-SVs	NAIBR
50–1k	Benchmark	3671		
	Total call	11 495	0	10
	True positive	2954	0	0
	False positive	8541	0	10
	False negative	717	3584	3584
	Precision	25.7%	NAN	NAN
	Recall	80.5%	0%	0%
1k–10k	Benchmark	499		
	Total call	602	0	157
	True positive	317	0	119
	False positive	285	0	38
	False negative	182	499	380
	Precision	52.7%	NAN	75.8%
	Recall	63.5%	0%	23.8%
>10k	Benchmark	29		
	Total call	105	35	218
	True positive	6	6	24
	False positive	99	29	194
	False negative	23	23	5
	Precision	5.7%	17.1%	11.0%
	Recall	20.7%	20.7%	82.8%

## Funding

This research was supported by Vanderbilt University Development Funds (FF_300033), the Joint Initiative for Metrology in Biology (JIMB; National Institute of Standards and Technology) and Research Grant Council Early Career Scheme (HKBU 22201419).

*Conflict of Interest:* There is NO Competing Interest.

## Supplementary Material

vbab007_Supplementary_Data
