# Supplementary material for: Aquila_stLFR: diploid genome assembly based structural variant calling package for stLFR linked-reads
Source: Bioinform Adv. 2021 Jun 16;1(1):vbab007. doi: 10.1093/bioadv/vbab007 (PMC9710574; doi:10.1093/bioadv/vbab007)
Supplement: vbab007_Supplementary_Data [file vbab007_supplementary_data.pdf]

Aquila_stLFR: diploid genome assembly based structural variant calling package for stLFR linked-reads

Supplemental Material

Hybrid assembly of stLFR and 10X linked-reads and SV detection from the hybrid mode of Aquila_stLFR

We calculated the parameters of the stLFR library as follows: $C = 48, C_R = 0.2X, C_F = 237.6X, \mu_{FL} = 30.1kb$ (Supplementary Table 2). C represents raw coverage, C_R represents average coverage of short reads per fragment, C_F represents the average physical coverage of the genome by long DNA fragments, and μ_{FL} represents mean unweighted DNA fragment length. By comparing the stLFR library parameters with those of the 10X ones in Supplementary Table 2 ($C = 93, C_R = 0.12X, C_F = 760.0X, \mu_{FL} = 44.8kb$), low μ_{FL} and raw coverage C from stLFR could be responsible for the lower contiguity of stLFR assemblies (N50 22.4kb for stLFR vs. N50 96.4kb for 10X linked-reads in Supplementary Table 2). Our recent studies (Zhang et al., 2019, 2020) also suggest that the optimal raw coverage of 10X linked-reads for diploid assembly and SV detection from assemblies is at least $56X$ and the quality of diploid assembly benefits from longer average fragment length. Furthermore, the stLFR library used a shorter, $100bp$ length of paired short reads, which was a disadvantage for local assembly compared to the $150bp$ length of paired short reads used by 10x linked-reads sequencing.

To increase the raw coverage C and μ_{FL}, we used the hybrid mode in Aquila_stLFR to assemble both stLFR and 10X linked-reads (GiAB NA24385 stLFR library + NA24385 10X linked-reads library 5 in Zhou et al., 2021). Both raw coverage C and C_F increased almost linearly by the combination of the stLFR and 10X libraries (Supplementary Table 2). Mean fragment length μ_{FL} also increased compared to the stLFR library. Hybrid assembly further improved the continuity of diploid contigs (N50) compared to both stLFR and 10X linked-reads libraries (Supplementary Table 2), and the improvement was more significant for the stLFR library. We also saw that for SV detection, the F1 score of the hybrid mode increased by 12.4%, 23.3%, and 27.7% respectively for three different sizes of deletions compared to the stLFR library (Supplementary Table 3), and 20.7% and 4.78% for two different sizes of insertions (Supplementary Table 4). Compared to the 10X library, the hybrid mode increased 1.7%, 0.1% and 4.3% respectively for three different sizes of deletions (Supplementary Table 3), and 0.8% and 0.5% for two different sizes of insertions (Supplementary Table 4). The hybrid mode could further remove more false positive SVs to improve the overall performance for 10X linked-reads. In conclusion, the hybrid mode outperformed both stLFR and 10X linked-reads libraries, and the improvement was most significant for stLFR linked-reads by increasing both recall and precision. The overall F1 score also improved by filtering more false positives relative to 10X linked-reads.

Our study provides a guideline for future stLFR library preparation to achieve significant improvement in diploid assembly and SV detection.

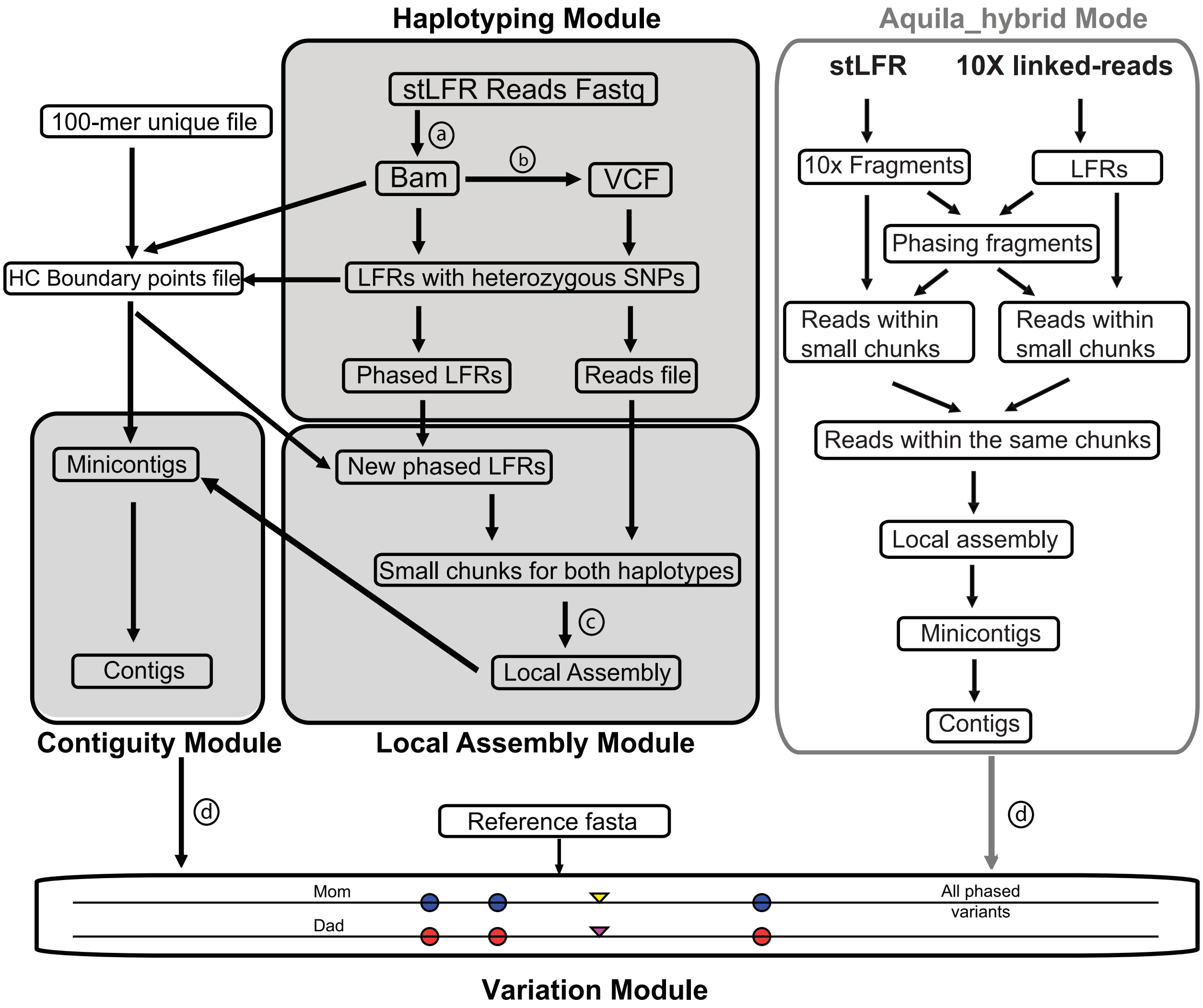

Supplemental Figure 1: Pipeline of Aquila_stLFR, a reference-assisted diploid-resolved genome assembly for stLFR. Input files: FASTQ file, BAM file and VCF file. a: Bwa-mem; b: FreeBayes; c: SPAdes; d: minimap2 and paftools.

```
@CL100066606L2C016R059_503217   BX:Z:540_839_548
AAAACACACTTTTATTTTATTTTATTTTATTTATTTAATTTAATTTAATTTAATTTTATTTTATTTTATTTATTTTATTTTATTTTATTTTATTTTTTTG
+
A@;,<EEEE<6B=EEDAFEEEC@FECE8DE@BB=ECCCAFBA-E?ABD>BEF3EE=@332E@EB>E.EABE:D=EDAA6ECFEBE=AEEFFB@EBE?>E9
@CL100066606L2C016R059_503217   BX:Z:540_839_548
CCTGTAGTCCCAGCTACTCAGGAGGCTGAGGCAGGAGAATGGCGTGAACCCAGGAGACAGAGCTTGCAGTGAGCTGAGATAGCGTCACTGCACTCCAGCC
+
EEFFE<FEDEFFFEFEEEF?EEAF8FFD:?EFEFEAD2D6<EFEFF@FFEEFED?EAEDF3DFFFFECFFE,EFFFAFEFDCFEFFDCFFCDFFFD=<<A
@CL100066606L2C016R091_430379   BX:Z:540_839_548
CTCACCCCTTCTACCATGTGAGGACGCAGCAAGAAGTCACCTTCTATAAATCAGGAAGAGATCGCTAACCATCCCCTGTATCTGCTAGTGCCTGGATCTT
+
F5F>EFFFD*FA<FED;F>DCFF==FFDEFFFFA=F8FFFF:EFAE4E-0<7<?FA<FBF'@AFF3B/=FA@FFF<=F(ECF8FF5FFAFE8@FF?DF-=
@CL100066606L2C016R091_430379   BX:Z:540_839_548
CATATAACCTCACCCACATAGAGAAATAAAATAACTAGGATCCAGATGTTCCTGCATAAACCCTCTTGATTCTTACCCATTCACATTCTCCAGAAATAAC
+
CFDEAB6FDFEEFDE<F>4AE4=+4@FBD;:BE>EE;EE29AF:F1DF5/EE8'E;6<5%CEECBB.F0>3EBBFB;F>A3E?F;C.8<BF2&&@9;E,E
@CL100066606L2C017R026_292022   BX:Z:540_839_548
CCTTCACTTTCAGGTTTTAATGTATTCAACTCATTTGGATAAATACCAAGGAGCACAGCCTCTTCTTTTTTATTATTGAGCAATCTCAAATGCATCTCTG
+
FEEEFF.EF<FFFDEEF@E@@EFFCBFD?EEECEDAFBFCD@FEFF?F:E>?FEFD>3EFCCE455FDCE?F9DEE>D8:EF8DEFEEFEEFF=6FEFDE
@CL100066606L2C017R026_292022   BX:Z:540_839_548
CCCTCCTTTGGGAGATGCTGCTCTTGAAGTAGAAGAAAAGTGCTAACTGGAAATTCTTTGAAACCTCACTTTCTCATTGTCAGCTGGTCAAAAGAAAACA
+
FFFFF>EFFFFDFFFFFFEFFFFFFEFEE:EFFFFFCFFDFFFFFF@FFFEC56EFFFDDF>9=FFFFFFFE@<FF'E>FBFCFFDGFE,F/@@E=D4EFF
```

Supplemental Figure 2: A screen shot of stLFR fastq reads, it shows three pairs of short reads. Before performing reads alignment by bwa-mem with “-C” flag, add barcode “BX:Z:barcode” at the header of each read.

```
CL200047468L1C006R038_39389     147     chr1    9997    0       100M    =       10013   -84     CAGATAACCCTAACCCTAACCCTAACCCTAACCCTAACCCT
AACCCTAACCCTAACCCTAACCCTAACCCTAACCCTAACCCTAACCCTAACCCTAACCC     9CFEFGFAFG7FGFEFEGFDFFG6E@FFG?FFFFGDGFFFFFFFFFGEFFFFFGFEFFGG@FF@GGFFFFGGF
FF?FGDFFGGFFFFFEGFG;FFFA<FF     NM:i:1  MD:Z:1C98       AS:i:98 XS:i:96 BX:Z:586_902_1136       RG:Z:12878:LibraryNotSpecified:1:unknown_
fc:0-43F6A2D2
CL200047469L1C011R060_87815     147     chr1    9997    0       100M    =       10013   -84     CAGATAACCCTAACCCTAACCCTAACCCTAACCCTAACCCT
AACCCTAACCCTAACCCTAACCCTAACCCTAACCCTAACCCTAACCCTAACCCTAACCC     FFDFFFCGFGEFGFFFFG1FFGFCFFFFGFGFFGE<FFFFFFGFFFGFGFFFEGG>FFFGFFFFFFFFFFFFF
FFFEFF>FFGFFFFFEFFFFFFFFFFF     NM:i:1  MD:Z:1C98       AS:i:98 XS:i:96 BX:Z:353_1476_263       RG:Z:12878:LibraryNotSpecified:1:unknown_
fc:0-64378858
CL100066606L2C006R022_551816    147     chr1    9997    0       100M    =       10007   -90     CAGATAACCCTAACCCTAACCCTAACCCTAACCCTAACCCT
AACCCTAACCCTAACCCTAACCCTAACCCTAACCCTAACCCTAACCCTAACCCTAACCC     FFFECDDFFFFFFDEFE>FECFFE6FEDFFGFEFF>FDBFFCFFFDFFFFDFFFFFFFFE6FFFFFFFCEFFF
FDEFFFFFFFFFFFDGFFEFFFFFFEC     NM:i:1  MD:Z:1C98       AS:i:98 XS:i:96 BX:Z:0_0_0      RG:Z:12878:LibraryNotSpecified:1:unknown_fc:0-43F
6A2D2-5F69628
CL200047469L1C009R005_5964      163     chr1    9998    0       100M    =       10005   108     CCATAACCCTAACCCTAACCCTAACCCTAACCCTAACCCTA
ACCCTAACCCTAACCCTAACCCTAACCCTAACCCTAACCCTAGCCCTAACCCTAACCCT     5E&F=?.)<@E1BD2DCF7*76FE<2<.E)6<-54<)(4?<-7D75F5BEED>>8=BEEF83;8?E4A0C-AA
*8=E1A,ECB%:1AFFC7F@BC03EDB     NM:i:2  MD:Z:1G81A16    AS:i:93 XS:i:92 BX:Z:46_1232_9  RG:Z:12878:LibraryNotSpecified:1:unknown_fc:0-515
43204
CL100066606L2C017R018_461042    99      chr1    9998    0       100M    =       10044   146     CCATAACCCTAACCCTAACCCTAACCCTAACCCTAACCCTA
ACCCTAACCCTAACCCTAACCCTAACCCTAACCCTAACCCTAACCCTAACCCTAACCCT     >:)GG@EFFGFD0F?EGFFGGEFFGFGBFFCGFBGCFFFFFEEC<GFB=FFFFFF7DFGGFDBFGDFDGEFFD
GGBE@FFEDF5:FGFG@BFGB7EFGFF     NM:i:1  MD:Z:1G98       AS:i:98 XS:i:97 BX:Z:584_924_493        RG:Z:12878:LibraryNotSpecified:1:unknown_
fc:0-64717E9D
```

Supplemental Figure 3: A screen shot of five reads from BAM file. Each read contains the barcode field “BX:Z:barcode”.

insertion		Aquila_stLFR
50-1k	Benchmark	
	Total call	1,311
	True Positive	1,099
	False Positive	212
	False Negative	3,591
	Precision	83.8%
	Recall	23.4%
1k-10k	Benchmark	
	Total call	6
	True Positive	2
	False Positive	4
	False Negative	726
	Precision	33.3%
	Recall	0.27%

Supplemental Table 1: Genome-wide insertion evaluation for GiAB NA24385 stLFR library against GIAB NA24385 benchmark.

Source	Raw Coverage $C(X)$	C_F (X)	C_R (X)	μ_{FL} (kb)	Contig N50(kb)	Contig NA50(kb)	Diploid Ratio
GiAB (stLFR)	48	238	0.20	30.1	23.3	22.4	2.03
L5 from Zhou et al., 2021 (10X)	93	760	0.12	44.8	98.4	96.4	2.04
Hybrid library (stLFR +10X)	134	959	0.14	41.9	100.4	100.3	2.06

Supplemental Table 2: Parameters of stLFR, 10X linked-reads library, and the hybrid library for sample NA24385. C_F: physical (fragment) coverage; C_R: read coverage per fragment; C: raw coverage $C \geq C_F * C_R$, μ_{FL}: mean fragment length. All four parameters for three libraries were generated through their hg19 BAM files. Assembly metrics of contig N50/NA50 and diploid fraction were generated by Quast (Gurevich et al., 2013). Diploid ratio is the total number of aligned bases in the NA24385 assembly divided by the total number of aligned bases in the hg19 reference genome.

Deletion		Aquila_stLFR (stLFR)	Aquila (10X)	Aquila (stLFR + 10X)
50-1k	Benchmark	3671		
	Total call	11,495	9,804	9,226
	True Positive	2,954	3,349	3,317
	False Positive	8,541	6,455	5,909
	False Negative	717	322	354
	Precision	25.7%	34.2%	36.0%
	Recall	80.5%	91.2%	90.4%
	**F1**	**39.0%**	**49.7%**	**51.4%**
1k-10k	Benchmark	499		
	Total call	602	452	445
	True Positive	317	384	382
	False Positive	285	68	63
	False Negative	182	115	117
	Precision	52.7%	85.0%	85.8%
	Recall	63.5%	77.0%	76.6%
	**F1**	**57.6%**	**80.8%**	**80.9%**
>10k	Benchmark	29		
	Total call	105	45	31
	True Positive	6	12	11
	False Positive	99	33	20
	False Negative	23	17	18
	Precision	5.7%	26.7%	35.5%
	Recall	20.7%	41.4%	37.9%
	**F1**	**9.0%**	**32.4%**	**36.7%**

Supplemental Table 3: Genome-wide deletion evaluation for the NA24385 stLFR library, NA24385 10X linked-reads library 5 (L5) from Zhou et al., 2021, and the hybrid library (stLFR + 10X linked-reads libraries) against GIAB NA24385 benchmark.

Insertion		Aquila_stLFR (stLFR)	Aquila (10X)	Aquila (stLFR + 10X)
50-1k	Benchmark	4690		
	Total call	1,311	4,012	3,549
	True Positive	1,099	2,457	2,359
	False Positive	212	1,555	1,190
	False Negative	3,591	2,233	2,331
	Precision	83.8%	61.2%	66.5%
	Recall	23.4%	52.4%	50.3%
	**F1**	**36.6%**	**56.5%**	**57.3%**
1k-10k	Benchmark	728		
	Total call	6	21	24
	True Positive	2	18	20
	False Positive	4	3	4
	False Negative	726	710	708
	Precision	33.3%	85.7%	83.3%
	Recall	0.27%	2.47%	2.75%
	**F1**	**0.54%**	**4.81%**	**5.32%**

Supplemental Table 4: Genome-wide insertion evaluation for the NA24385 stLFR library, NA24385 10X linked-reads library 5 (L5) from Zhou et al., 2021, and the hybrid library (stLFR + 10X linked-reads libraries) against GIAB NA24385 benchmark.

References

Gurevich A, Saveliev V, Vyahhi N, Tesler G (2013) QUAST: quality assessment tool for genome assemblies. Bioinformatics 29:1072–1075 http://www.ncbi.nlm.nih.gov/pubmed/23422339.

Zhang L, Zhou X, Weng Z, Sidow A (2019) Assessment of human diploid genome assembly with 10x Linked-Reads data. Gigascience 8:1–11.

Zhang L, Zhou X, Weng Z, Sidow A (2020) De novo diploid genome assembly for genome-wide structural variant detection. NAR Genomics Bioinforma 2:1–10.

Zhou X, Zhang L, Weng Z, Dill DL, Sidow A (2021) Aquila enables reference-assisted diploid personal genome assembly and comprehensive variant detection based on linked reads. Nat Commun 12 http://dx.doi.org/10.1038/s41467-021-21395-x.
